# The over-optimistic portrayal of life-supporting treatments in newspapers and on the Internet: a cross-sectional study using extra-corporeal membrane oxygenation as an example

**DOI:** 10.1186/1472-6939-15-59

**Published:** 2014-08-01

**Authors:** Yen-Yuan Chen, Likwang Chen, Yu-Hui Kao, Tzong-Shinn Chu, Tien-Shang Huang, Wen-Je Ko

**Affiliations:** 1 Department of Social Medicine, National Taiwan University College of Medicine, No. 1, Rd. Ren-Ai sec. 1, Taipei 10051, Taiwan; 2 Department of Medical Education, National Taiwan University Hospital, No. 7, Rd. Chong-Shan S, Taipei 10002, Taiwan; 3 Institute of Population Health Sciences, National Health Research Institutes, 35 Keyan Road, Zhunan, Miaoli County 35053, Taiwan; 4 Department of Sociology, Iowa State University, 103 East Hall, Ames, IA 50011, USA; 5 Department of Primary Care Medicine, National Taiwan University College of Medicine, No. 1, Rd. Ren-Ai sec. 1, Taipei 10051, Taiwan; 6 Department of Internal Medicine, National Taiwan University Hospital, No. 7, Rd. Chong-Shan S, Taipei 10002, Taiwan; 7 Department of Surgery, National Taiwan University College of Medicine, No. 1, Rd. Ren-Ai sec. 1, Taipei 10051, Taiwan; 8 Department of Traumatology, National Taiwan University Hospital, No. 7, Rd. Chong-Shan S, Taipei 10002, Taiwan

**Keywords:** Life-supporting treatment, Extra-corporeal membrane oxygenation, Media, Internet, Newspaper

## Abstract

**Background:**

Extra-corporeal membrane oxygenation has been introduced to clinical practice for several decades. It is unclear how internet and newspapers portray the use of extra-corporeal membrane oxygenation. This study were: (1) to quantify the coverage of extra-corporeal membrane oxygenation use in newspapers and on the Internet; (2) to describe the characteristics of extra-corporeal membrane oxygenation users presented in newspaper articles and the Internet web pages in comparison with those shown in extra-corporeal membrane oxygenation studies in Taiwan; and (3) to examine the survival rates of extra-corporeal membrane oxygenation users presented in newspaper articles and the Internet web pages in comparison with those in Taiwan and in the Extracorporeal Life Support Registry Report International Summary for January 2014.

**Methods:**

All issues of Taiwan’s four major newspapers from 2006 to 2010 were reviewed. In October 2011, a search of Internet web pages was performed based on the subjects of “yeh-ko-mo” (extra-corporeal membrane oxygenation in Traditional Chinese), “ECMO”, and “extra-corporeal membrane oxygenation.” All the Internet web pages and newspaper articles recounting the use of extra-corporeal membrane oxygenation were reviewed. The information, such as patient characteristic and the status at hospital discharge, was collected.

**Results:**

The survival rate of extra-corporeal membrane oxygenation use shown on the Internet (83.97%) was significantly higher than all the survival rates reported in Taiwan’s literature (*p* < .01) and in the Extracorporeal Life Support Registry Report International Summary for January 2014 (*p* < .01). In addition, the survival rate of extra-corporeal membrane oxygenation use shown in newspapers (61.54%) was significantly higher than the average survival rate (43%) reported in Taiwan’s literature, the pediatric average survival rate (51%), and the adult average survival rate (47%) in the Extracorporeal Life Support Registry Report International Summary for January 2014.

**Conclusions:**

Internet and newspapers both showed over-optimistic survival to hospital discharge for patients sustained by extra-corporeal membrane oxygenation. Internet was more likely to provide optimistic information for aggressive life-supporting treatments such as extra-corporeal membrane oxygenation than newspapers as indicated by survival to hospital discharge.

## Background

Extra-corporeal membrane oxygenation (ECMO) is a technique for providing cardiac and respiratory support to patients by using a modified heart-lung machine. ECMO can support life for days to weeks, permitting treatment and recovery during severe cardiac or respiratory failure. According to the Extracorporeal Life Support Registry Report International Summary for January 2014 (ECLS 2014), the annual number of patients supported by ECMO increased from 1,644 in 1990 to 4,357 in 2013. From 1990 to 2013, 58,842 patients received ECMO for cardiac or respiratory failure, or received ECMO-assisted cardiopulmonary resuscitation (CPR), with 35,307 (60%) surviving to hospital discharge. Of the 58,842 patients supported by ECMO, 33,412 (56.78%) were neonatal patients, who have the highest rates of immediate survival after ECMO use, and the highest rates of survival to hospital discharge, compared with adult and pediatric patients. Of the 5,146 adult patients who received ECMO because of respiratory failure, 2,905 (56.45%) survived to hospital discharge. Of the 4,042 patients who received ECMO because of cardiac failure, 1,636 (40.48%) survived to hospital discharge. The average rate of adult ECMO patients surviving to hospital discharge was 46.96% [1].

Taiwan has become one of the major countries for ECMO use. In 2010, 1,126 Taiwanese patients received ECMO support [2]. In six Taiwanese studies reporting the survival of ECMO users with cardiac failure, the survival rates at hospital discharge ranged from 27.78% to 64% [3-8], with an approximate average survival rate of 44.03%. A study by Chen et al. in 2011 reported a 31.58% survival rate of ECMO users with respiratory failure (Table 1) [6].

**Table 1:** Studies conducted in Taiwan for reporting the survival rates for extra-corporeal membrane oxygenation use

	**Location**	**Primary reason**	**Age (Mean ± SD)**	**Gender (F/M)**	**Alive/Dead at hospital discharge**	**Survival rate**
**Chung SY et al., 2012 [**[8]**]**	A	CF	51.8 ± 20.5	30/104	57/77	42.50%
		RF	NA	NA	NA	NA
**Hsu KH et al., 2011 [**[7]**]**	B	CF	29.7 ± 18.7	46/29	48/27	64%
		RF	NA	NA	NA	NA
**Chen YC et al., 2011 [**[6]**]**	A	CF	47 ± 2^a^	40/62	36/44	45%
		RF	NA	NA	6/13	31.58%
**Chou NK et al., 2010 [**[5]**]**	B	CF	42.3 ± Unknown^a^	5/35	21/19	52.50%
		RF	NA	NA	NA	NA
**Hsu PS et al., 2010 [**[4]**]**	C	CF	63.0 ± 15.7	15/36	17/34	33.33%
		RF	NA	NA	NA	NA
**Ko WJ et al., 2002 [**[3]**]**	B	CF	56.8 ± 15.9	28/48	20/52	27.78%
		RF	NA	NA	NA	NA

*Abbreviations list*: *CF* cardiac failure, *RF* respiratory failure, *SD* standard deviation, *F/M* female/male, *NA* not applicable, *A* medical center A, *B* medical center B, *C* medical center C.

^a^The values were the median of patients’ ages.

The mass media, such as newspapers and the Internet, can have a substantial influence on the public’s attitudes and behaviors. For example, newspapers and the Internet provide readers with information on advances in medical technology, and the use of life-supporting treatments (LST). The information in newspapers and on the Internet may thus influence the decision-making of patients/family members regarding the request for an aggressive LST such as ECMO [9].

The purpose of our study was to examine the portrayal of ECMO use by Taiwan’s four major newspapers and the Internet web pages written in traditional Chinese. The specific aims were to: (1) quantify the coverage of ECMO use on newspapers and the Internet; (2) describe the characteristics of ECMO users presented in newspaper articles and the Internet web pages in comparison with those shown in ECMO studies in Taiwan; and (3) determine the survival rates of ECMO users presented in newspaper articles and the Internet web pages in comparison with those in ECMO studies in Taiwan and in the ECLS 2014.

## Methods

### Selection of stories

Some ECMO patients were featured in newspapers only (*patients in newspapers*), or on the Internet only (*patients on the Internet*). Those both featured on newspapers and the Internet were *patients both in newspapers and on the Internet. Patients* were defined as the ECMO patients either reported in newspapers or in the Internet. Therefore, *patients* are equal to the total of *patients in newspapers*, *patients on the Internet*, and *patients both in newspapers and on the Internet*.

**Figure 1:**
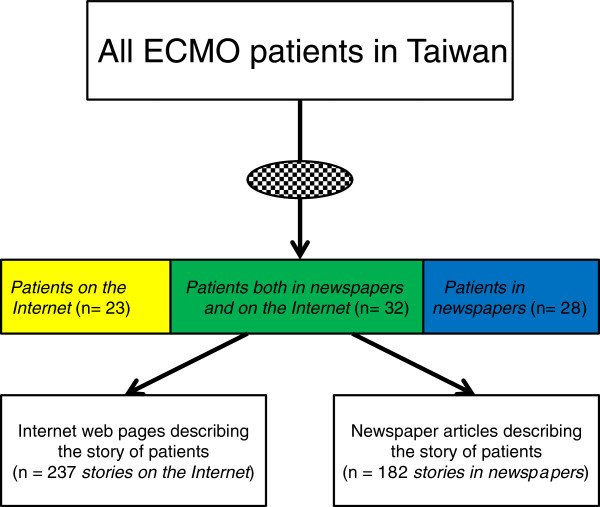
**The extra-corporeal membrane oxygenation users selected and reported by the Internet and newspapers.** Abbreviations list: ECMO=Extra-corporeal Membrane Oxygenation. “” means that some stories of ECMO patients were selectively reported on the Internet web pages and in newspaper articles. ““ were the 23 ECMO patients who were only reported on Internet web pages. ““ were the 32 ECMO patients who were both reported on Internet web pages and newspaper articles. ““ were the 28 ECMO patients who were only reported in newspaper articles.

A newspaper article describing a case of ECMO patient was a *story in newspaper*. An Internet web page describing a case of ECMO patient was a *story on the Internet*. Each single *patient in newspaper* or *patient on the Internet* could appear in several newspaper articles and Internet web pages, thus resulting in numerous *stories in newspapers* and *stories on the Internet* (Figure 1).

All issues of Taiwan’s four major newspapers, the Liberty Times, the Apple Daily, the China Times and the United Daily News, from 2006 to 2010, were searched and reviewed, and the headlines and texts of the newspaper articles on ECMO use were counted as a *story in newspaper*. In October 2011, a search of Internet web pages was performed using “yeh-ko-mo” (ECMO in traditional Chinese medicine), “ECMO,” and “extra-corporeal membrane oxygenation” as search terms. Each single Internet web page recounting ECMO use was counted as a *story on the Internet*.

### Data collection

For each *story on the Internet* and *story in newspaper*, the following information was collected: age, sex, the location of the hospital of ECMO use (rural or urban area), the level of the hospital (medical center or non-medical center, as classified by Taiwan Joint Commission on Hospital Accreditation), the duration of ECMO use, the duration of intensive care unit stay, the duration of hospital stay, the status at hospital discharge, and the primary reason for ECMO use.

### Statistical analysis

The continuous variables of *patients*, *patients on the Internet*, *patients in newspapers*, *stories on the Internet*, and *stories in newspapers* were compared with those from studies on ECMO use in Taiwan published in journals indexed in the Sciences Citation Index [3-8], and the ECLS 2014 [1], using one-sample *t*-tests. The associations between the characteristics of *patients* and *patients on the Internet*, between *patients* and *patients in newspapers*, and between *patients* and *stories*, were evaluated using Student’s *t*-tests or Chi-squared tests. The survival rates of *patients*, *patients on the Internet*, *patients in newspapers*, *stories on the Internet*, and *stories in newspapers* were compared with those derived from the ECMO studies in Taiwan and the ECLS 2014, using one-sample *t*-tests. All statistical analyses were performed using the software package STATA MP 11.0 for Windows PC.

## Results

### Comparison of characteristics

Overall, we collected 419 *stories* (83 *patients*): 237 *stories* (55 *patients on the Internet*) from the Internet web pages and 182 *stories* (60 *patients on newspapers*) from the newspaper articles (Figure 1 and Table 2). Thirty-two of the ECMO *patients* appeared both in the newspaper articles and on the Internet web pages. Of the 182 *stories in newspapers* (*patients in newspapers*) collected from the four major newspapers, 12 (4), 23 (7), 75 (22), 42 (16), and 30 (11) *stories in newspapers* (*patients in newspapers*) were from the years 2006, 2007, 2008, 2009, and 2010, respectively.

**Table 2:** Comparison of the characteristics and the rates of surviving to hospital discharge

	***Stories on the Internet *** **(n = 237)**	***p*** **value**^**a**^	***Patients on the Internet *** **(n = 55)**	***p*** **value**^**b**^	***Patients *** **(n = 83)**	***p*** **value**^**c**^	***Patients in newspapers *** **(n = 60)**	***p*** **value**^**d**^	***Stories in newspapers *** **(n = 182)**
**Age**^**e**^	33.37 ± 21.62	0.71	34.65 ± 23.75	0.47	31.68 ± 22.72	0.63	29.88 ± 21.31	0.73	28.75 ± 21.75
**Length of ECMO use by day**^**f**^	15.66 ± 26.95	0.34	11.66 ± 18.79	0.74	10.57 ± 16.08	0.55	12.37 ± 17.95	0.86	12.87 ± 18.91
**Length of ICU stay by day**^**g**^	16.90 ± 16.91	0.70	15.64 ± 17.55	0.60	13.67 ± 15.80	0.93	13.36 ± 13.83	0.75	14.21 ± 14.04
**Length of hospital stay by day**^**h**^	38.69 ± 50.61	0.64	43.13 ± 64.68	0.60	36.86 ± 56.41	0.57	31.49 ± 36.88	0.97	31.72 ± 38.19
**Gender**^**i**^		0.51		0.53		0.55		0.95	
Male	146 (63.76%)		35 (68.63%)		50 (63.29%)		35 (58.33%)		107 (58.79%)
Female	83 (36.24%)		16 (31.37%)		29 (36.71%)		25 (41.67%)		75 (41.21%)
**Hospital location**		0.22		0.34		0.99		0.98	
Urban	190 (80.17%)		40 (72.73%)		54 (65.06%)		39 (65%)		118 (64.84%)
Rural	47 (19.83%)		15 (27.27%)		29 (34.94%)		21 (35%)		64 (36.16%)
**Level of hospital**^**j**^		0.12		0.55		0.90		0.69	
Medical center	168 (70.89%)		33 (60%)		45 (54.88%)		33 (55.93%)		106 (58.89%)
Non-medical center	69 (29.11%)		22 (40%)		37 (45.12%)		26 (44.07%)		74 (41.11%)
**Primary reason for ECMO use**		0.43		0.89		0.95		0.99	
Cardiac	127 (53.59%)		26 (47.27%)		40 (48.19%)		30 (50%)		93 (51.10%)
Pulmonary	95 (40.08%)		23 (41.82%)		32 (38.55%)		23 (38.33%)		68 (37.36%)
Others	15 (6.33%)		6 (10.91%)		11 (13.25%)		7 (11.67%)		21 (11.54%)
**Status at hospital discharge**		0.31		0.28		0.68		0.48	
Alive	199 (83.97%)		43 (78.18%)		58 (69.88%)		40 (66.67%)		112 (61.54%)
Dead	38 (16.03%)		12 (21.82%)		25 (30.12%)		20 (33.33%)		70 (38.46%)

*Abbreviations list:ECMO* extra-corporeal membrane oxygenation, *ICU* intensive care unit.

Interpretations of “Status at hospital discharge”: 58 (69.88%) *patients* survived to hospital discharge, and 43 (78.18%) *patients on the Internet* survived to hospital discharge. The *p* value (0.28) is not significant; 43 (78.18%) *patients on the Internet* survived to hospital discharge, and 199 (83.97%) *stories on the Internet* survived to hospital discharge. The *p* value (0.31) is not significant.

^a^The *p* value of the comparisons between *stories on the Internet* (n = 237) and *patients on the Internet* (n = 55).

^b^The *p* value of the comparisons between *patients on the Internet* (n = 55) and *patients* (n = 83).

^c^The *p* value of the comparisons between *patients* (n = 83) and *patients in newspapers* (n = 60).

^d^The *p* value of the comparisons between *patients in newspapers* (n = 60) and *stories in newspapers* (n = 182).

^e^“Age”: *stories on the Internet* = 6 missing values; *patients on the Internet* = 3 missing values; *patients* = 3 missing values.

^f^“Length of ECMO use by day”: *stories on the Internet* = 20 missing values; *patients on the Internet* = 8 missing values; *patients* = 11 missing values; *patients in newspapers* = 5 missing values; *stories in newspapers* = 9 missing values.

^g^“Length of ICU stay by day”: *stories on the Internet* = 92 missing values; *patients on the Internet* = 22 missing values; *patients* = 35 missing values; *patients in newspapers* = 24 missing values; *stories in newspapers* = 66 missing values.

^h^“Length of hospital stay by day”: *stories on the Internet* = 71 missing values; *patients on the Internet* = 15 missing values; *patients* = 20 missing values; *patients in newspapers* = 13 missing values; *stories in newspapers* = 28 missing values.

^i^“Gender”: *stories on the Internet* = 8 missing values; *patients on the Internet* = 4 missing values; *patients* = 4 missing values.

^j^“Level of hospital”: *patients* = 1 missing value; *patients in newspapers* = 1 missing value; *stories in newspapers* = 2 missing values.

Because the average ages of the ECMO patients in the studies by Chen et al. and Chou et al. were unavailable [5,6], and because some of the ECMO patients in the study by Chen et al. were also included in the study by Chung et al., we excluded these two studies from our analyses. In the remaining four studies [3,4,7,8], the average age of the 336 patients was 49.70 years. Of these patients, 119 (35.42%) were women. The rates of survival to hospital discharge for the ECMO users ranged from 27.78% to 64%, with an average survival rate of 42.77%.

The average ages of the 419 *stories* and 83 *patients* were 31.34 (±21.77) years and 31.68 (±22.72) years, respectively. These ages were significantly lower than the average ages of the patients in the four studies (*p* < .01). We noted that 38.44% of the 419 *stories* and 36.71% of the 83 *patients* were women. These values showed non-significant differences from the percentages of women in the four studies.

Myocarditis (121 *stories*, 22 *patients*), acute respiratory distress syndrome (86 *stories*, 13 *patients*), and acute myocardial infarction (44 *stories*, 12 *patients*) were the three leading primary causes of the need for ECMO. *Patients* with longer durations of ECMO use, longer durations in an intensive care unit, or cardiac failure as the primary reason for initiating ECMO were likely to have a greater number of *stories both in newspapers and on the Internet* than other patients. The numbers of *stories* and *patients* surviving to hospital discharge were 311 (74.22%) and 58 (69.88%), respectively.

### Comparison of survival rates

We compared the survival rate of the 83 *patients* with that of the 55 *patients on the Internet* and with that of the 60 *patients in newspapers*. We also compared the survival rate of the 55 *patients on the Internet* with that of the 237 *stories on the Internet*, and compared the survival rate of the 60 *patients in newspapers* with that of the 182 *stories in newspapers* (Tables 2 and 3). Although the average survival rate of the 55 *patients on the Internet* was 8.3% higher than that of the 83 *patients* (*p* = .28), and the average survival rate of the 237 *stories on the Internet* was 5.79% higher than that of the 55 *patients on the Internet* (*p* = .31), these differences were not statistically significant. Similarly, the difference between the survival rate of the 83 *patients* and that of the 60 *patients in newspapers* (*p* = .68), and the difference between the survival rate of the 60 *patients in newspapers* and that of the *stories in newspapers* (*p* = .48), were not statistically significant (Table 2).

**Table 3:** **Comparison of the characteristics and the rates of surviving to hospital discharge between “ *Patients *” and “ *Stories *”**

	***Stories on the Internet *** **(n = 237)**	***p*** **value**^**a**^	***Patients *** **(n = 83)**	***p*** **value**^**b**^	***Stories in newspapers *** **(n = 182)**
**Age**^**c**^	33.37 ± 21.62	0.55	31.68 ± 22.72	0.32	28.75 ± 21.75
**Length of ECMO use by day**^**d**^	15.65 ± 26.95	0.13	10.57 ± 16.08	0.37	12.87 ± 18.91
**Length of ICU stay by day**^**e**^	16.90 ± 16.91	0.24	13.67 ± 15.80	0.83	14.21 ± 14.04
**Length of hospital stay by day**^**f**^	38.69 ± 50.61	0.81	36.86 ± 56.41	0.44	31.72 ± 38.19
**Gender**^**g**^		0.94		0.50	
Male	146 (63.76%)		50 (63.29%)		107 (58.79%)
Female	83 (36.24%)		29 (36.71%)		75 (41.21%)
**Hospital location**		< 0.01		0.97	
Urban	190 (80.17%)		54 (65.06%)		118 (64.84%)
Rural	47 (19.83%)		29 (34.94%)		64 (36.16%)
**Level of hospital**^**h**^		< 0.01		0.54	
Medical center	168 (70.89%)		45 (54.88%)		106 (58.89%)
Non-medical center	69 (29.11%)		37 (45.12%)		74 (41.11%)
**Primary reason for ECMO use**		0.14		0.88	
Cardiac	127 (53.59%)		40 (48.19%)		93 (51.10%)
Pulmonary	95 (40.08%)		32 (38.55%)		68 (37.36%)
Others	15 (6.33%)		11 (13.25%)		21 (11.54%)
**Status at hospital discharge**		< 0.01		0.19	
Alive	199 (83.97%)		58 (69.88%)		112 (61.54%)
Dead	38 (16.03%)		25 (30.12%)		70 (38.46%)

*Abbreviations list:ECMO* extra-corporeal membrane oxygenation, *ICU* intensive care unit.

Interpretations of “Status at hospital discharge”: 58 (69.88%) *patients* survived to hospital discharge, and 199 (83.97%) *stories on the Internet* survived to hospital discharge. The *p* value (<0.01) is significant. That is, the survival of ECMO *patients on the Internet* is over-optimistic.

^a^The *p* value of the comparisons between *patients* (n = 83) and *stories on the Internet* (n = 237).

^b^The *p* value of the comparisons between *patients* (n = 83) and *stories in newspapers* (n = 182).

^c^“Age”: *stories on the Internet* = 6 missing values; *patients* = 3 missing values.

^d^“Length of ECMO use by day”: *stories on the Internet* = 20 missing values; *patients* = 11 missing values; *stories in newspapers* = 9 missing values.

^e^“Length of ICU stay by day”: *stories on the Internet* = 92 missing values; *patients* = 35 missing values; *stories in newspapers* = 66 missing values.

^f^“Length of hospital stay by day”: *stories on the Internet* = 71 missing values; *patients* = 20 missing values; *stories in newspapers* = 28 missing values.

^g^“Gender”: *stories on the Internet* = 8 missing values; *patients* = 4 missing values.

^h^“Level of hospital”: *patients* = 1 missing value; *stories on the Internet* = 1 missing value; *stories in newspaprts* = 2 missing values.

Subsequently, we compared the survival rate of the 83 *patients* with that of the 237 *stories on the Internet*, and with that of the 182 *stories on newspapers* (Table 3). The average survival rate of the 237 *stories on the Internet* was significantly higher than that of the 83 *patients* (*p* < .01). Although the average survival rate of the 182 *stories in newspapers* was 8.34% lower than that of the 83 *patients*, this difference was not statistically significant (*p* = .19). We identified that the Internet web pages were more likely to report a higher rate of survival to hospital discharge following ECMO use than newspaper articles.

**Figure 2:**
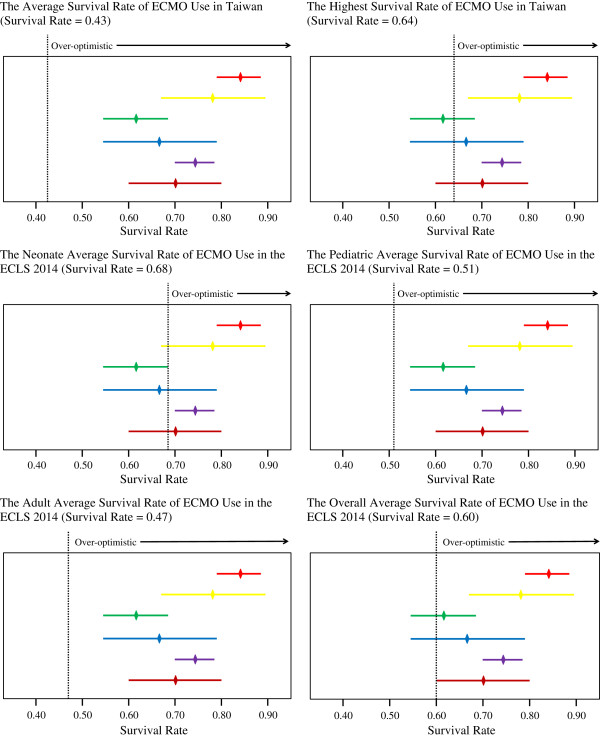
**The comparison of survival rates of extra-corporeal membrane oxygenation users with those reported in the literature.** Abbreviations list: ECMO=Extra-corporeal Membrane Oxygenation; ECLS 2014= Extracorporeal Life Support Registry Report International Summary January 2014. “” is the mean and 95% confidence interval of the survival rate of *stories on the Internet*. “” is the mean and 95% confidence interval of the survival rate of *patients on the Internet*. “” is the mean and 95% confidence interval of the survival rate of *stories in newspapers*. “” is the mean and 95% confidence interval of the survival rate of *patients in newspapers*. “” is the mean and 95% confidence interval of the survival rate of *stories*. “” is the mean and 95% confidence interval of the survival rate of *patients*. “Over-optimistic” means that if the mean and 95% confidence interval are located entirely at the right side of the dotted line, the survival rate of ECMO users shown on the media is significantly higher than the survival rate obtained from the literature. For example, based on the left upper part, the  is located entirely at the right side of the dotted line, “The Average Survival Rate of ECMO Use in Taiwan” (Survival Rate=0.43). Therefore, the survival rate of *stories* on the Internet is over-optimistic as compared to “The Average Survival Rate of ECMO Use in Taiwan”.

We compared the survival rates of the 83 *patients*, 419 *stories*, 55 *patients on the Internet*, 60 *patients in newspapers*, 237 *stories on the Internet*, and 182 *stories in newspapers* with those of the patients in the ECMO studies in Taiwan and the ECLS 2014 (Figure 2). The average survival rate of the 419 *stories* (74.22%) was significantly higher than the highest survival rate in the four studies and the ECLS 2014 (*p* < .01). Although the survival rate of the 83 *patients* (69.88%) was higher than the highest survival rate reported in Taiwan (64%), this difference did not achieve statistical significance (*p* = .25). The average survival rate of the 83 *patients* showed non-significant differences from the average survival rate of all ECMO patients (60%) reported in the ECLS 2014 (*p* = .05).

The survival rate of the 237 *stories on the Internet* was significantly higher than the survival rates reported in the ECMO studies in Taiwan (*p* < .01) and in the ECLS 2014 (*p* < .01). However, the survival rate of the 182 *stories in newspapers* was not significantly higher than the highest survival rate of ECMO use in Taiwan (*p* = .50), and the average survival rate of ECMO use for all patients in the ECLS 2014 (*p* = .67) (Figure 2 and Table 3).

The majority of the 419 *stories* and 83 *patients* were adult patients, whereas the majority of the patients from whom data were collected in the ECLS 2014 were neonatal patients. Therefore, we also compared the survival rates presented in newspaper articles and Internet web pages with the average survival rate of the adult ECMO patients reported in the ECLS 2014 (Figure 2). The survival rates of the 419 *stories* and 83 *patients* were both significantly higher than the average survival rate of the adult ECMO patients (46.96%) reported in the ECLS 2014 (*p* < .01).

## Discussion

Novel LSTs such as ECMO typically attract considerable media attention in Taiwan [9]. In this study, we investigated the medical information on ECMO use presented in newspapers and on Internet web pages. We identified that: 1) Internet web pages were more likely to reproduce or duplicate the survival of ECMO patients than newspaper articles; and 2) the survival rates for ECMO use reported in newspapers and on Internet web pages were both over-optimistic as compared to those published in journals and the ECLS 2014.

### Media and life-supporting treatment

In 1996, Diem et al. investigated the depiction of CPR in three popular television medical dramas in the United States, and observed that the medical information, such as the immediate survival rate of patients who received CPR, provided to the public by the medical dramas was over-optimistic [10]. However, three other studies conducted to evaluate the CPR survival rates shown in television medical dramas failed to identify significant differences from the survival rates provided in the literature [11-13]. The findings of the present study, based on newspapers and Internet web pages, are consistent with Diem and colleagues’ findings from television medical dramas, in that medical information provided by newspapers and the Internet to the public is over-optimistic as indicated by the survival rate of the patients sustained using LST, and may mistakenly convince the public that an aggressive LST (such as CPR and ECMO) can rescue patients from all life-threatening conditions. Although the portrayal of the survival of patients receiving aggressive LST on television medical dramas remains controversial, it is apparent that such media outlets can substantially influence the public [14-17].

A study by Moynihan et al. investigated the coverage of three medications in the US news, observing that 60% of the news stories reported the potential benefits of the medications, whereas 47% of the news stories reported the potential harms. The study concluded that the coverage of the three drugs focused predominantly on their benefits [18]. Bartlett et al. reported that medical journals generally provide equal coverage of the positive and negative results of medical research, whereas newspapers are more likely to publish the negative results [19]. For example, one study concluded that jogging is significantly associated with beneficial effects on health [20] and another study showed that the risk of acute myeloid leukemia is significantly increased in cockpit crews [21]. Newspapers would tend to report the results of the second study rather than the first. Our study results showed that the survival rate of the 60 *patients in newspapers* and 182 *stories in newspapers* were both significantly higher than the average survival rates in the four ECMO studies in Taiwan, which suggested that newspapers are likely to provide over-optimistic medical information on ECMO use as a LST.

For ECMO use shown on the Internet web pages, we found two ways that the medical information was over-optimistic: First, writers or bloggers of Internet web pages were more likely to select the alive ECMO users than the dead, as indicated by the significantly higher survival rate of the 55 *patients on the Internet* than the highest survival rates of ECMO use (*p* = .01) in the four studies in Taiwan; Second, writers or bloggers were more likely to reproduce or duplicate the alive ECMO users shown by other Internet web pages than the dead ECMO users, as indicated by the higher survival rate of the 237 *stories on the Internet* than that of the 55 *patients on the Internet* (*p* = .31). Writers or bloggers of the Internet web pages may not have particular interests in reporting, reproducing or duplicating optimistic outcomes of ECMO use. Instead, they may report, reproduce or duplicate stories of their own personal interests or those which are effective in attracting readers’ attention.

Medical decisions to request aggressive LST is frequently discussed in the field of medical ethics. Physicians assist with a patient’s or family member’s medical decision-making by providing medical information and suggestions based on scientific and humanistic principles, and with respect for patient autonomy. Patients usually make medical decisions depending on the medical information and suggestions given by the physicians, personal values, personal preferences, and past medical experiences [22]. Therefore, the over-optimistic survival rate of ECMO users reported by the newspaper articles and Internet web pages may potentially influence patients’ and family members’ personal values and personal preferences, thus encouraging them to request ECMO being performed on themselves or their relatives while death is imminent.

None of the laws associated with clinical practice in Taiwan (e.g., Physicians Act [23], Medical Care Act [24], Hospice Palliative Care Act [25]) forces physicians to provide aggressive LST which is considered inappropriate to patients even if patients/family members request them. Therefore, physicians theoretically can decline the request of inappropriate LST based on their professional judgment. However, if the family members are influenced by the over-optimistic survival rate of the inappropriate LST users reported in newspaper articles and Internet web pages, and thus strongly request the inappropriate LST to be performed on the patients, physicians, usually in fear of litigation or the burdensome process of litigation [26,27], are more likely to perform that LST without carefully deliberating its clinical indications.

### Strengths and limitations

This study evaluated the medical information on ECMO use presented in newspaper articles and Internet web pages. According to the 2010 Annual Report of Mass Media by Shih Hsin University College of Journalism and Communications [28], 97.3%, 74%, and 71.3% of adults in Taiwan, aged between 15 and 64, acquired health-related information by watching television, reading newspapers, and searching the Internet, respectively. Readers of the Liberty Times, the Apple Daily, the United Daily News, and the China Times constituted 16.9%, 15.9%, 7.7%, and 6.1% of Taiwanese people who read newspapers, respectively [29]. Medical information on ECMO use in the four major newspapers and on Internet web pages may, thus, influence a large proportion of the Taiwanese population.

Our study has two major limitations. First, our results might not be applicable to other newspapers in Taiwan and elsewhere in the world, or to other Internet web pages not using traditional Chinese language. Second, we obtained our data for analyses from newspapers in Taiwan and the Internet web pages. The stories of ECMO use reported by newspaper articles and Internet web pages might not contain all the variables we needed in this study. Therefore, some variables in this study inevitably had missing values.

## Conclusions

Newspapers and the Internet have the potential to influence patients' knowledge and attitudes toward medical decision-making by providing over-optimistic medical information through the following ways: First, the mass media tend to attract the public’s attention by reporting the positive outcome of an important breakthrough in clinical medicine; Second, the mass media tend to report patients who survive to hospital discharge, rather than those who die during hospital stay; Third, the survived patients and their stories are more likely to be duplicated in newspapers and on Internet web pages than those who die during hospital stay. Newspaper readers and Internet users may, therefore, mistakenly believe that ECMO can usually rescue patients from all life-threatening conditions. However, ECMO, similar to other aggressive LST such as CPR, is ethically appropriate to be initiated on patients with reversible diseases, not on those with irreversible diseases. Future research should examine the influence of the over-optimistic information provided by the two means of mass media on medical decision-making for requesting aggressive LST such as ECMO use, as well as the attitudes physicians have toward the use of inappropriate LST requested by patients with over-optimistic information provided via mass media.

## Abbreviations

CPR: Cardiopulmonary resuscitation; ECMO: Extra-corporeal membrane oxygenation; ECLS 2014: Extracorporeal Life Support Registry Report International Summary for January 2014; LST: Life-supporting treatments.

## Competing interests

The authors declare that they have no competing interests.

## Authors’ contributions

YC (Chen YY) carried out the literature review, study design, statistical analyses, manuscript drafting, and manuscript editing. LC (Chen L) carried out the statistical analyses and manuscript editing. YK (Kao YH) helped with the literature review, data collection and data comparison. TC (Chu TS) helped with manuscript editing. TH (Huang TS) helped with study design and manuscript editing. WK (Ko WJ) participated in manuscript editing. All authors read and approved the final manuscript.

## Pre-publication history

The pre-publication history for this paper can be accessed here:

http://www.biomedcentral.com/1472-6939/15/59/prepub
